# Heart 3D: echocardiographic and anatomical features of the tricuspid valve in a heterogeneous population with severe regurgitation—implications for edge-to-edge procedure suitability

**DOI:** 10.3389/fcvm.2025.1637158

**Published:** 2025-08-22

**Authors:** Jan Sobieraj, Adam Rdzanek, Agnieszka Kapłon-Cieślicka, Zenon Huczek, Mariusz Tomaniak, Ewa Ostrowska, Adam Piasecki, Ewa Pędzich, Piotr Scisło

**Affiliations:** First Chair and Department of Cardiology, Medical University of Warsaw, Warsaw, Poland

**Keywords:** tricuspid valve, tricuspid valve repair, echocardiography, three-dimensional (3D), transesophageal (TEE), anatomy, transcatheter

## Abstract

**Aim:**

To assess the incremental value of real-time three-dimensional (3D) transesophageal echocardiography (TEE) in visualizing tricuspid valve (TV) anatomy for procedural planning and guidance of transcatheter edge-to-edge repair (TEER) in cases of severe tricuspid regurgitation (TR).

**Materials and methods:**

An observational study was conducted on 54 patients with severe TR. The visualization of the TV leaflets during systole was graded semiquantitatively using predefined criteria: 0 points—no visible leaflet border or tissue; 1.25—border only; 2—border and <50% tissue; 3—border and >50% tissue. Each of the three leaflets was evaluated independently in both two-dimensional (2D) and 3D TEE, with a maximum cumulative score of 9. Two thresholds were established: ≥4.5 points as the primary endpoint for adequate visualization for TEER planning and ≥6 points as the secondary endpoint indicating sufficient quality for a detailed morphological assessment.

**Results:**

In 3D TEE, 77.8% of patients achieved the primary endpoint, and 68.5% reached the secondary threshold. In comparison, 2D TEE enabled 74.1% and 42.6% of patients to meet these respective thresholds. Although the difference in achieving the primary endpoint was not statistically significant (*p* = 0.82), 3D TEE significantly outperformed 2D TEE in enabling a detailed morphological evaluation (*p* = 0.012). No significant differences were noted in the visualization quality of the anterior vs. septal leaflets with 3D TEE (67.4% vs. 65.4%, *p* = 0.800). For the posterior leaflet, 3D TEE provided superior visualization compared with the 2D TEE (*p* = 0.0008), while still supporting procedural suitability in a comparable proportion of patients (85.4% vs. 89.8%, *p* = 0.400). Acoustic shadowing from the interatrial septum and aortic root accounted for 92% of inadequate visualizations.

**Conclusion:**

In this observational study, real-time 3D TEE proved feasible for assessing tricuspid valve anatomy and visualization quality in patients with severe TR who were considered for TEER. Compared with 2D TEE, 3D TEE offered an improved visualization of the posterior leaflet and provided adequate image quality for procedural planning in most patients. Moreover, a statistically significant advantage was observed for 3D TEE over 2D TEE in providing image quality sufficient for a detailed morphological evaluation.

## 1 Introduction

Severe tricuspid regurgitation (TR) is linked to increased mortality. While traditional surgical methods are the standard treatment, many patients face high surgical risks because of age and comorbidities. The development of less invasive transcatheter techniques offers a promising alternative, allowing high-risk patients to receive effective treatment while reducing the complications associated with open-heart surgery (1–5). Transcatheter tricuspid valve interventions include a clip-based leaflet transcatheter edge-to-edge repair (TEER), transcatheter annuloplasty, and bioprosthetic valve implantation (6–11). Regardless of the interventional method, accurate imaging is crucial for procedural success. Both fluoroscopy and echocardiography are essential for real-time evaluation of cardiac structures and implanted devices during catheter-based procedures. While fluoroscopy provides a precise visualization of catheters and device positioning, echocardiography uniquely enables a real-time assessment of soft tissue anatomy and hemodynamic function. Importantly, echocardiography offers these advantages without exposing operators or patients to ionizing radiation, thereby enhancing procedural safety (12, 13). Assessing the tricuspid valve is particularly challenging due to its unique morphological variability, varying leaflet thickness, and the presence of significant acoustic barriers that impede optimal imaging (14–17). The specificity of tricuspid valve imaging could potentially be improved with three-dimensional (3D) echocardiography, which enables visualization of the region of interest with minimal probe manipulation, although it requires greater expertise from the echocardiographer (18). The American Society of Echocardiography guidelines endorse both the midesophageal and transgastric imaging planes for the two-dimensional (2D) transesophageal echocardiographic (TEE) evaluation of the tricuspid valve (19). However, in the context of intraprocedural TEER guidance, maintaining the probe in a stable midesophageal position is often preferred. This approach not only minimizes the risk of mechanical trauma associated with repeated probe manipulation but also enhances imaging consistency throughout the procedure (20–22). Importantly, one of the key advantages of real-time 3D TEE is its ability to provide a comprehensive, *en face* visualization of the tricuspid valve from the midesophageal position. This reduces the need for multiple probe adjustments and facilitates both procedural planning and real-time guidance, supporting the feasibility and practical utility of 3D TEE in the TEER setting. While data on the application of 3D TEE in transcatheter tricuspid valve interventions were formerly limited, there has been exponential growth in research publications in recent years, driven by advancements in volumetric imaging and the clinical adoption of implantable devices (23–25). Thus, this study seeks to determine the enhanced utility of real-time 3D TEE in both procedural planning and intraprocedural guidance during TEER for severe TR.

## 2 Materials and methods

### 2.1 Study design and population

A total of 54 consecutive patients with severe TR who underwent TEE at a single high-volume center were prospectively included in the study. Patients were prospectively recruited between October 2024 and May 2025. The study protocol received approval from the institutional review board, and informed consent was obtained from all participants.

### 2.2 Echocardiographic evaluation

**Figure 1:**
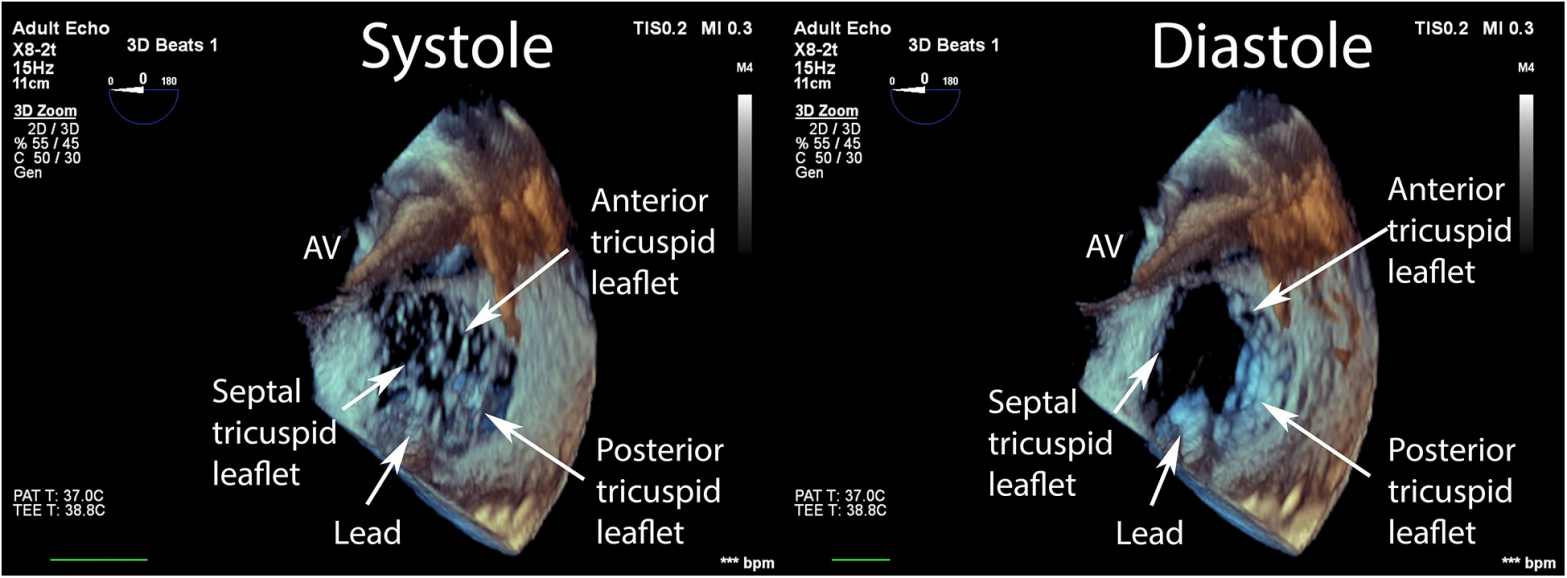
*En face* view of the tricuspid valve in real-time, three-dimensional transesophageal echocardiography. Visualization of sufficient quality for transcatheter tricuspid valve repair guidance includes the components of the valve: (i) annulus; (ii) septal, anterior, posterior leaflets; (iii) fragments of the subvalvular apparatus and anatomical landmarks: (iv) fragment of the aortic valve annulus, (v) fragment of the intra-atrial septum, and (vi) vena cava superior.

**Figure 2:**
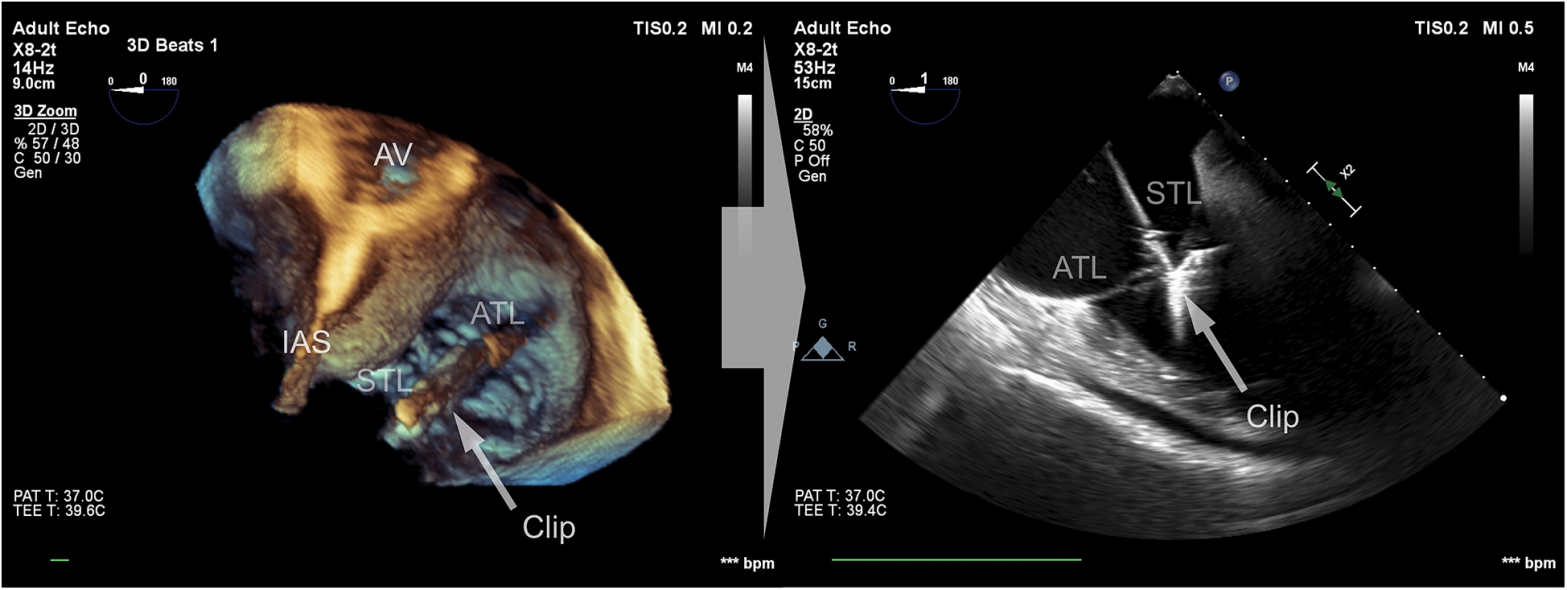
Cross-sectional 2D view of the device and leaflets at the point of implantation based on a 3D view after confirming the perpendicularity between the device and the leaflets.

Data were collected using a Philips Epiq CVx rev. 4.0 with a transesophageal x8-2t probe and stored on a Philips Xcelera PACS ver. 3.1. A complete preprocedural echocardiographic assessment of the tricuspid valve was assumed to include the following: (i) an *en face* planar 3D visualization of the tricuspid valve borders and leaflets during systole to provide spatial orientation during the introduction of a delivery system and device implantation (Figure 1); and (ii) a cross-sectional 2D (inflow–outflow right ventricle) view through the device and leaflet at the implantation point (Figure 2).

In our protocol, TEE was performed with the patient under conscious sedation using a split dose of midazolam. Image acquisition began by positioning the probe at the midesophageal level, which is considered optimal for tricuspid valve visualization in a four-chamber view at 0 degrees. By standardizing image acquisition at the midesophageal level, the protocol ensures that both 2D and 3D datasets are directly comparable, thereby enabling a fair assessment of the incremental value provided by 3D TEE. Next, a single-beat, real-time, 3D TEE zoom volume image depicting the components of the tricuspid valve and anatomical landmarks (Figure 1) was acquired. Then, the optimal sector shape, representing a compromise between size and temporal resolution, was selected as the reference for procedural planning and guidance.

The recorded material was reviewed on external workstations with the Philips QLAB 15.5 software with a plug-in by a single experienced echocardiographer. Basic echocardiographic parameters presented in Table 1 were calculated. A real-time 3D TEE zoom volume image was rotated to depict the tricuspid valve complex from the right atrial roof in an *en face* fashion, and the proper visualization of each leaflet in the systolic phase was semiquantitatively described. The tricuspid valve leaflets were assessed according to the following predetermined criteria: (i) neither leaflet border nor leaflet tissue was identified—0 points; (ii) only a leaflet border, but no leaflet tissue was identified—1.25 points; (iii) a leaflet border and partial leaflet tissue (<50%) were identified—2 points; and (iv) a leaflet border and leaflet tissue (>50%) were identified—3 points. Points scored by each leaflet were summed, and the maximum possible result was 9 points. This subjective scoring system for assessing the visualization quality of the tricuspid valve leaflets was originally adapted from the four-point scale described by Anwar et al. and subsequently modified based on the framework proposed by Kucken et al. (26, 27). In the current study, the scale was further refined by redefining the lower boundary for poor imaging quality to 0 points and introducing an additional increment of 0.25 to the 1-point category. This adjustment was made to underscore the critical importance of precise border visualization, particularly in the context of TEER procedures.

**Table 1:** Baseline characteristics and comparison of tricuspid valve annulus parameters in patients stratified according to the quality of real-time three-dimensional transesophageal echocardiography.

Parameters	Visualization of sufficient quality for TEER		*P*-value
	Yes (*n* = 42)	No (*n* = 12)	
Age (years)	64.3 (CI 95% 59.9–69.8)	65.6 (CI 95% 60.8–70.4)	0.77
Men	67%	83%	0.27
TEE indication			
Arrhythmia	58.3%	57.1%	0.94
Structural	47.1%	42.9%	0.94
Pacemaker leads	34%	32%	0.95
Antero-posterior dimension (mm)	37.6 (CI 95% 27.7–55.6)	39.7 (CI 95% 24.3–81.0)	0.99
Latero-medial dimension (mm)	43.8 (CI 95% 23.5–56.5)	38.4 (CI 95% 27.4–50.4)	0.49
Annulus height (mm)	5.5 (CI 95% 2.4–11.6)	8.4 (CI 95% 2.1–12.3)	0.052
Tricuspid valve 3D perimeter (mm)	123 (CI 95% 90.9–170.5)	135.8 (CI 95% 98.4–152.9)	0.29
Tricuspid valve 2D perimeter (mm)	116.7 (CI 95% 87.7–163.9)	121.8 (CI 95% 87.2–147.5)	0.64
Tricuspid valve 2D area (mm^2^)	1,042.2 (CI 95% 567.5–2,560.9)	995.2 (CI 95% 553.9–1,636.9)	0.84
Tricuspid valve 3D area (mm^2^)	1,104.2 CI 95% (569.4–2,594.4)	1,054.8 (CI 95% 631.5–1,688.6)	0.65

2D, two-dimensional; 3D, three-dimensional; TEE, transesophageal echocardiography; TEER, transcatheter edge-to-edge repair.

As highlighted in the literature, even anatomically suitable patients may have tricuspid leaflets that are exceptionally thin and challenging to visualize. While thin leaflet tissue is less optimal for secure device grasping, the leaflet border or coaptation zone serves as the principal anatomical landmark for effective device anchoring during TEER (28).

The primary endpoint, visualization of sufficient quality for the guidance of TEER, was met when (i) the total score was ≥4.5 points; (ii) at least one leaflet scored 2 points; and (iii) the remaining leaflets scored at least 1.25 points each.

The secondary endpoint, visualization of sufficient quality for a detailed morphologic evaluation of the tricuspid valve, was met when (i) the total score was ≥6 points; and (ii) all leaflets scored at least 2 points each.

The thresholds utilized in the present study were adapted from the methodology described by Kucken et al. (27). In their study, optimal image quality was defined as achieving a score of 15–18 on an 18-point scale, while scores between 10 and 15 indicated sufficient image quality. Given that the maximum achievable score on our proposed scale is 9 points, we have proportionally adjusted the thresholds to more accurately stratify patients into groups of “sufficient quality for TEER guidance” (≥4.5 points) and “detailed morphologic evaluation of the tricuspid valve” (≥6 points).

To enhance the discriminatory power of our scoring system, we introduced an additional criterion for the primary endpoint: at least one leaflet must score a minimum of 2 points, with the remaining leaflets each achieving at least 1.25 points. This modification prevents scenarios in which only two leaflets attain high visualization scores, thereby ensuring a more balanced assessment of the entire tricuspid valve. For the secondary endpoint, the requirement that each leaflet achieve at least 2 points further reduces the likelihood that two leaflets with perfect scores disproportionately influence the overall evaluation.

The potential limitations of this modified, subjective scoring system are discussed in detail in the limitations section.

### 2.3 Statistical analysis

Continuous variables were expressed as the mean ± standard deviation or median and interquartile range and were compared with Student's *t*-test or the Mann–Whitney *U* test as appropriate. Chi-square or Fisher's exact tests were used to compare categorical variables, expressed as counts and percentages. For each individual leaflet, the differences between the 2D and 3D visualization scores were assessed for normality using the Shapiro–Wilk test. Based on the results of the Shapiro–Wilk test, the Wilcoxon signed-rank test was employed to compare the 2D and 3D visualization scores. Finally, the median scores for both 2D and 3D visualizations were calculated and compared to determine which method tended to produce higher-quality scores. Agreement between the measurements was also assessed using the Bland–Altman method. All probability values were two-tailed, and a *p*-value of < 0.05 was considered statistically significant. The data were processed using MedCalc 14.0 (MedCalc, Ostend, Belgium), Wizard 1.9, and R 4.4.3 with the nortest and effsize packages.

## 3 Results

**Figure 3:**
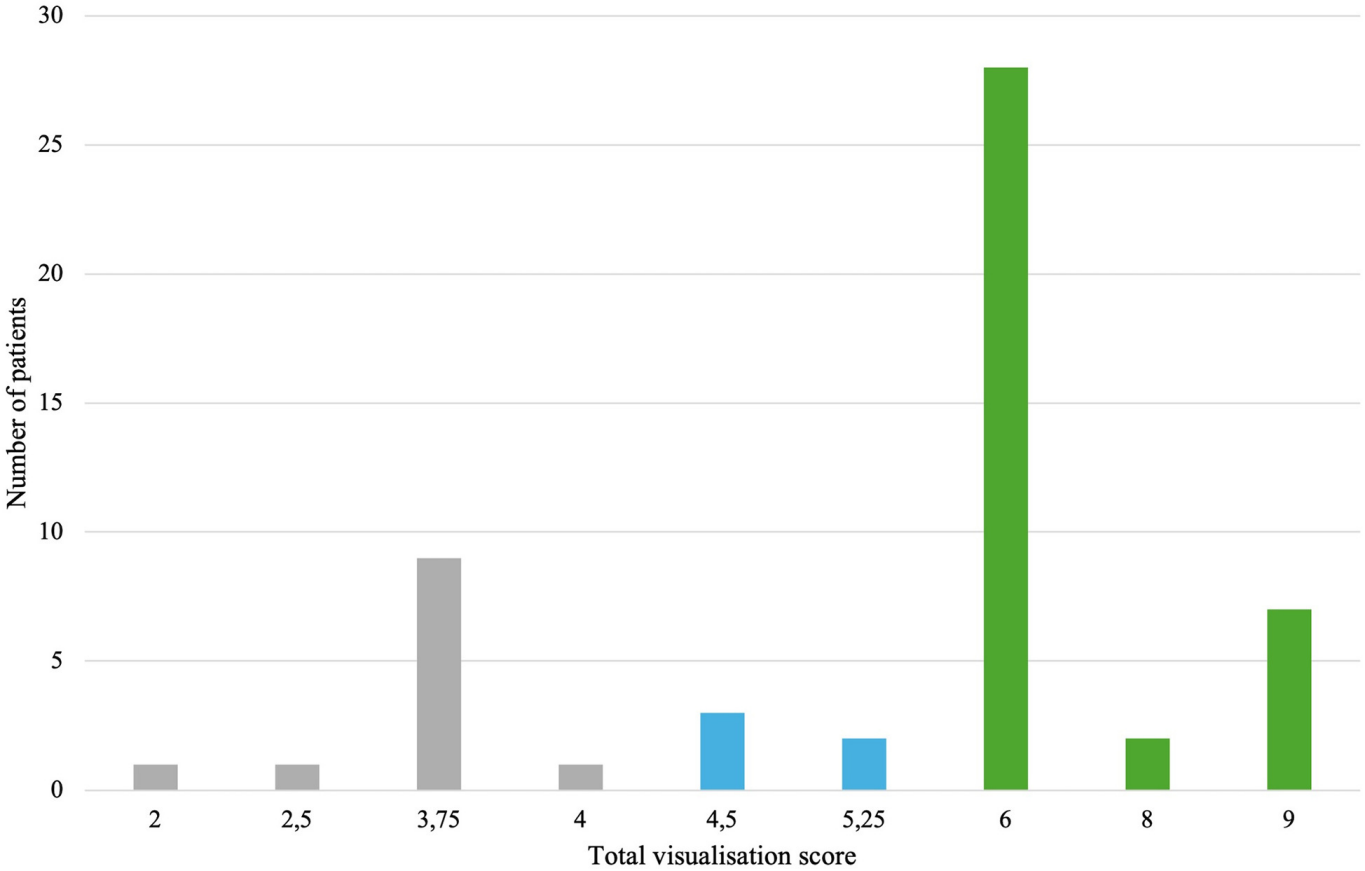
Distribution of total visualization scores for each patient in 3D TEE. Gray color—insufficient visualization quality for procedure guidance, blue color—adequate study quality for guiding TEER (primary endpoint), green color—adequate study quality for a detailed morphological evaluation (secondary endpoint). TEE, transesophageal echocardiography; TEER, transcatheter edge-to-edge repair.

**Figure 4:**
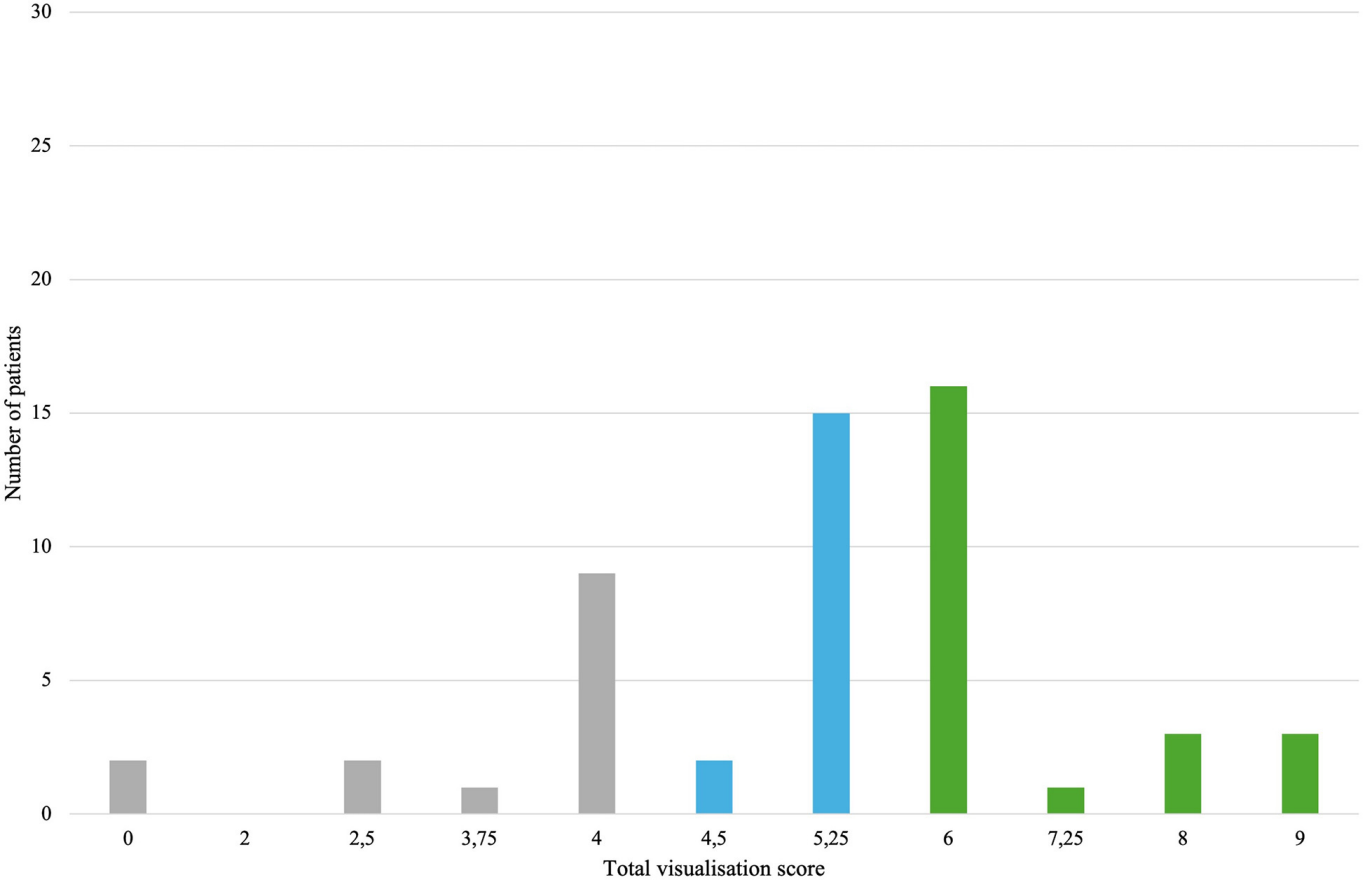
Distribution of total visualization scores for each patient in 2D TEE. Gray color—insufficient visualization quality for procedure guidance, blue color—adequate study quality for guiding TEER (primary endpoint), green color—adequate study quality for a detailed morphological evaluation (secondary endpoint). TEE, transesophageal echocardiography; TEER, transcatheter edge-to-edge repair.

In 3D TEE examinations, a visualization of sufficient quality for TEER guidance (the primary endpoint) was achieved in 42 patients (77.8%). The secondary endpoint, defined as quality suitable for a detailed morphologic evaluation of the valve, was achieved in 37 patients (68.5%) (Figure 3). In contrast, 2D TEE enabled 40 patients (74.1%) to reach the primary endpoint and 23 patients (42.6%) to achieve the secondary endpoint (Figure 4). While there was no statistically significant difference between 3D and 2D TEE in the proportion of patients achieving the primary threshold for adequate visualization (*p* = 0.82), there was a statistically significant difference between 3D and 2D TEE in achieving the higher threshold for the detailed morphological evaluation (*p* = 0.012). Furthermore, a comparison of echocardiographic parameters and tricuspid valve measurements (Table 1) showed no statistically significant differences between patients with and without sufficient TEE imaging quality.

**Figure 5:**
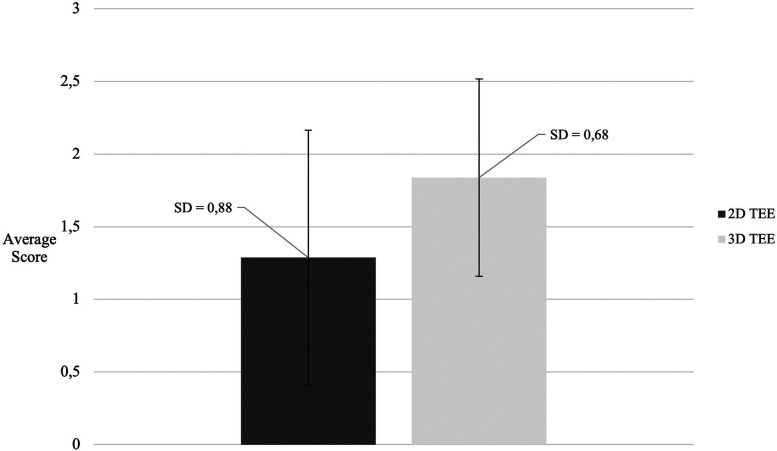
Comparison of posterior leaflet visualization scores between 2D and 3D TEE. TEE, transesophageal echocardiography.

The results of our study indicate that real-time 3D TEE provides sufficient visualization to support transcatheter intervention in 89.8% of patients for both the anterior and the septal leaflets. There was no statistically significant difference between these two leaflets in terms of visualization quality (*p* = 1.000). A detailed morphological evaluation was achievable in 67.4% of anterior leaflets and 65.4% of septal leaflets, with no significant difference observed between them (*p* = 0.800). The posterior leaflet was visualized with adequate quality for intervention in 85.4% of patients, which was comparable to the rates for the anterior and the septal leaflets (85.4% vs. 89.8%, *p* = 0.400). Importantly, a statistically significant difference in visualization scores between 2D and 3D echocardiography was found only for the posterior leaflet. Specifically, 3D TEE yielded significantly higher visualization scores for the posterior leaflet compared with 2D examination (*p* = 0.0008), as shown in Figure 5.

**Figure 6:**
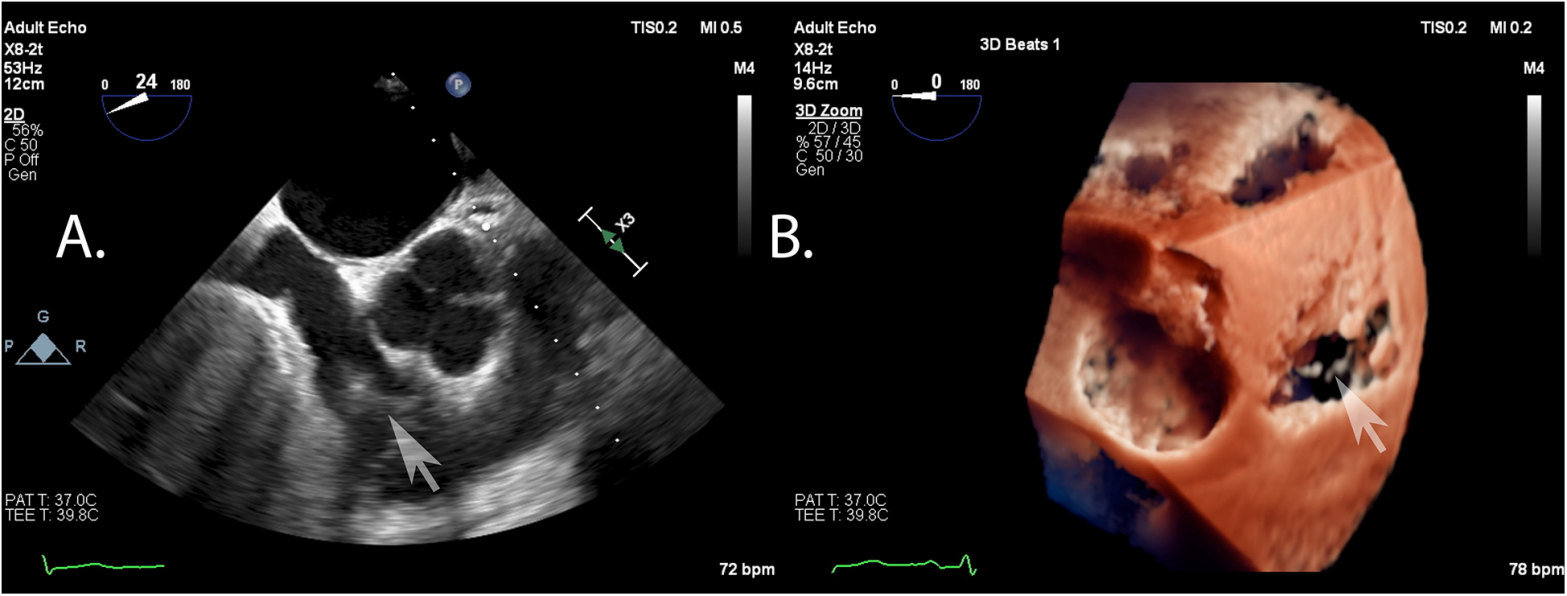
Example of typical artifacts precluding a proper visualization of the tricuspid valve: shadowing from the intra-atrial septum and signal attenuation as seen in the *en face* view of two- **(A)** and three-dimensional **(B)** echocardiography.

Acoustic shadowing from the intra-atrial septum and the aortic root, causing signal attenuation, was found to be a major factor associated with insufficient tricuspid valve visualization quality for TEER in 11 patients (92%) (Figure 6). In the remaining patient(8%), anatomical mismatch due to tricuspid ring displacement caused by the severe left ventricular dilatation was observed.

## 4 Discussion

Echocardiography is essential to guide transcatheter mitral and tricuspid interventions, in contrast to the procedures on aortic or pulmonary valves, where fluoroscopy usually provides sufficient visualization (3, 9, 10, 29–32). While initially supported with 2D protocols, the application of 3D echocardiography in both mitral and tricuspid procedures is expected to increase. Although data on real-time 3D TEE in TEER remain limited, we investigated its utility in planning and guiding the intervention in routine clinical practice.

Echocardiographic image quality plays a pivotal role in both the selection of candidates and the procedural success of TEER for TR. As highlighted in the GLIDE score study, image quality was identified as one of the five key anatomical and procedural determinants predicting successful outcomes. Limited echocardiographic visualization can hinder an accurate assessment of leaflet morphology, coaptation gaps, and jet location, which are essential for optimal device positioning and anchoring. The study conducted by Gercek et al. demonstrated that patients with good echocardiographic image quality had significantly higher rates of procedural success, defined as a substantial reduction in tricuspid regurgitation and the achievement of moderate or less residual TR. Conversely, suboptimal image quality was associated with increased procedural complexity and a higher likelihood of residual regurgitation (33). Therefore, a thorough preprocedural echocardiographic evaluation is critical not only for appropriate patient selection but also for maximizing the likelihood of therapeutic success with TEER-TR interventions.

It is well established that the tricuspid valve exhibits considerable morphological variability, with only approximately 54% of individuals having the classic three-leaflet configuration (34). In our study, we did not exclude patients with nonclassical leaflet anatomies. To maintain the clarity and interpretability of the scoring system, we elected not to subdivide the leaflets beyond the three principal anatomical components. In patients in whom a leaflet was anatomically divided or exhibited additional scallops or segments, it was assessed as a single leaflet within the corresponding main category. This approach was similarly applied to other variations in leaflet segmentation.

While this methodological choice simplifies the analysis and facilitates comparison across the cohort, it may not fully capture the spectrum of anatomical diversity present in the population. We acknowledge that this could potentially limit the granularity and real-world applicability of the scoring system, particularly in patients with atypical leaflet morphology.

The main finding of the present study is that real-time 3D TEE provides a good visualization of the tricuspid valve in patients with severe TR. Although the success rate for a visualization of sufficient quality for TEER did not differ between the respective tricuspid valve leaflets in 2D and 3D visualizations, the difference was not statistically significant for the posterior leaflet. Moreover, the individual leaflet analysis revealed a statistically significant difference in 2D and 3D visualization scores solely for the posterior leaflet. These observations may explain the low numbers of septal-posterior implantations, accounting for 20% of interventions, since 2D echocardiography remains the standard for diagnosis and monitoring. Antero-septal clipping remains the primary target in most TEER-TR interventions, as it offers superior visualization (35, 36). Further research is needed to determine whether the improved visualization of the posterior leaflet with 3D echocardiography could lead to a greater adoption of septal-posterior clipping.

### 4.1 Limitations

Although echocardiographic examinations were standardized, interobserver variability may still be present and pose a major limitation to this study. The consistency of the scoring system among different examiners has yet to be assessed. These findings should be validated in a larger patient population and further analyzed for both interobserver and intraobserver variability. In addition, the clinical relevance of the scoring thresholds has not been validated against procedural outcomes or compared directly with existing scales. External validation in an independent patient cohort is also lacking, which may limit the generalizability of our findings.

A further limitation of this study is the use of a standardized three-leaflet framework for scoring, regardless of the actual anatomical configuration of the tricuspid valve. By not subdividing additional leaflets, scallops, or accessory segments beyond the three principal categories, the analysis may not fully reflect the true morphological diversity encountered in clinical practice. As a result, the granularity and applicability of the scoring system may be reduced in patients with atypical or complex leaflet anatomy. Future studies should consider adapted scoring systems or subgroup analyses to better address the full range of leaflet variations described in the literature.

## 5 Conclusions

This observational study demonstrates that real-time 3D TEE is a feasible and effective imaging modality for the assessment of TV anatomy and visualization quality in patients with severe TR undergoing evaluation for TEER. The results indicate that 3D TEE provides a superior visualization of the posterior leaflet compared with 2D TEE and achieves adequate image quality for procedural planning and guidance in the majority of investigated cases. Moreover, a statistically significant improvement in image quality for a detailed morphological assessment was demonstrated with 3D TEE compared with 2D TEE. These findings support the incremental value of 3D TEE, particularly for achieving the higher-quality visualization necessary for a detailed morphological evaluation.

However, these findings should be interpreted within the context of the study's design and limitations. While 3D TEE frequently enabled sufficient visualization to support TEER guidance, this study does not establish 3D TEE as the definitive or sole primary imaging tool for all patients. Instead, the results support the utility and added value of 3D TEE as a complementary modality that can enhance intraoperative imaging and procedural feasibility in many, but not all, clinical scenarios.

Further prospective studies, ideally with larger, more diverse patient populations and comparative designs, are warranted to confirm these findings and to clarify the optimal role of 3D TEE in the procedural workflow for transcatheter tricuspid interventions.

## Data Availability

The raw data supporting the conclusions of this article will be made available by the authors, without undue reservation.
